# *trans*-Carbonyl­chloridobis(tri-*p*-tolyl­phosphine)rhodium(I) acetone solvate

**DOI:** 10.1107/S1600536808003528

**Published:** 2008-02-08

**Authors:** Fabio Lorenzini, Brian O. Patrick, Brian R. James

**Affiliations:** a Department of Chemistry, University of British Columbia, 2036 Main Mall, Vancouver, BC, Canada V6T 1Z1

## Abstract

The title compound, [RhCl(C_21_H_21_P)_2_(CO)]·C_3_H_6_O, was precipitated in trace yield from a reaction of RhCl(cod)(THP) with P(*p*-tol)_3_ in a 1:1 acetone-*d*_6_/CD_3_OD solution under a hydrogen atmosphere [*p*-tol = *p*-tolyl, THP = tris­(hydroxy­meth­yl)phosphine, P(CH_2_OH)_3_, and cod = 1,5-cyclo­octa­diene]. The complex displays a square-planar geometry around the Rh^I^ atom. The complex mol­ecules and the acetone mol­ecules are linked into a chain along the *a* axis by inter­molecular C—H⋯Cl and C—H⋯O hydrogen bonds.

## Related literature

For related literature, see: Beck *et al.* (1999 ▶, and references therein); Evans *et al.* (1990 ▶); Higham *et al.* (2004 ▶); Hoye *et al.* (1993 ▶); Lorenzini *et al.* (2007*a* ▶,*b* ▶, 2008*a* ▶,*b* ▶); Vallarino (1957 ▶).
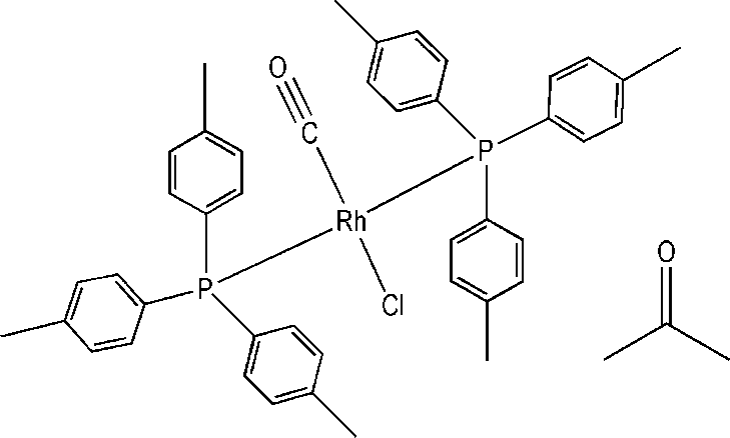


## Experimental

### 

#### Crystal data

[RhCl(C_21_H_21_P)_2_(CO)]·C_3_H_6_O
*M* *_r_* = 833.14
Triclinic, 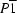

*a* = 10.784 (2) Å
*b* = 12.859 (3) Å
*c* = 17.086 (3) Å
α = 70.852 (7)°
β = 84.790 (7)°
γ = 71.012 (6)°
*V* = 2116.2 (7) Å^3^
*Z* = 2
Mo *K*α radiation
μ = 0.58 mm^−1^
*T* = 173 (2) K
0.25 × 0.10 × 0.07 mm

#### Data collection

Bruker X8 APEXII diffractometer
Absorption correction: multi-scan (*SADABS*; Bruker, 2003 ▶) *T*_min_ = 0.683, *T*_max_ = 0.960
31662 measured reflections
9909 independent reflections
6378 reflections with *I* > 2σ(*I*)
*R*_int_ = 0.063

#### Refinement

*R*[*F* ^2^ > 2σ(*F* ^2^)] = 0.047
*wR*(*F* ^2^) = 0.115
*S* = 1.00
9909 reflections
477 parameters
H-atom parameters constrained
Δρ_max_ = 0.48 e Å^−3^
Δρ_min_ = −0.44 e Å^−3^

### 

Data collection: *APEX2* (Bruker, 2006 ▶); cell refinement: *APEX2*; data reduction: *SAINT* (Bruker, 2005 ▶); program(s) used to solve structure: *SIR97* (Altomare *et al.*, 1999 ▶); program(s) used to refine structure: *SHELXL97* (Sheldrick, 2008 ▶); molecular graphics: *ORTEP-3 for Windows* (Farrugia, 1997 ▶); software used to prepare material for publication: *WinGX* (Farrugia, 1999 ▶).

## Supplementary Material

Crystal structure: contains datablocks I, global. DOI: 10.1107/S1600536808003528/ci2547sup1.cif

Structure factors: contains datablocks I. DOI: 10.1107/S1600536808003528/ci2547Isup2.hkl

Additional supplementary materials:  crystallographic information; 3D view; checkCIF report

## Figures and Tables

**Table:** 

C43—Rh1	1.812 (4)
P1—Rh1	2.3449 (9)
P2—Rh1	2.3283 (9)
Cl1—Rh1	2.3822 (9)

**Table:** 

C43—Rh1—P2	91.43 (10)
C43—Rh1—P1	90.55 (10)
P2—Rh1—P1	177.46 (3)
C43—Rh1—Cl1	178.12 (10)
P2—Rh1—Cl1	86.69 (3)
P1—Rh1—Cl1	91.33 (3)

**Table 2:** Hydrogen-bond geometry (Å, °)

*D*—H⋯*A*	*D*—H	H⋯*A*	*D*⋯*A*	*D*—H⋯*A*
C10—H10⋯O2^i^	0.95	2.38	3.302 (8)	163
C46—H46*A*⋯Cl1	0.98	2.81	3.773 (7)	168

Symmetry code: (i) 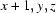.

## supplementary crystallographic information

### Comment

We have very recently reported the structure of *trans*-RhCl(CO)(PEtPh_2_)_2_, crystals of which precipitated serendipitously in trace yield from a reaction between PEtPh_2_ and RhCl(cod)(THP), where cod = 1,5-cyclooctadiene and THP = tris(hydroxymethyl)phosphine, P(CH_2_OH)_3_, in an acetone/MeOH solvent mixture under a hydrogen atmosphere (Lorenzini *et al.*, 2008*b*). Such reaction conditions with a phosphine of general formula P*RR*'_2_ (*R* = or ≠ *R*') lead to formation of the dihydrido complexes *cis*,*mer*-Rh(H)_2_Cl(P*RR*'_2_)_3_ (when *R*' = Ph, and *R* = Me or Cy) (Lorenzini *et al.*, 2008*a*) or, if the reaction is carried out under Ar, the phosphine–phosphinite derivatives RhCl(P*RR*'_2_)[*P*,*P*-*R*'(*R*)POCH_2_P(CH_2_OH)_2_] and trace amounts of the *trans*-RhCl(CO)(P*RR*'_2_)_2_ species (Lorenzini *et al.*, 2007*b*). The THP plays a critical role in formation of the dihydrides and the carbonyl complexes (Lorenzini *et al.*, 2008*a*); the CO ligand is thought to result from decarbonylation of formaldehyde (Beck *et al.*, 1999), which can be readily formed from transition metal–THP species (Higham *et al.*, 2004; Hoye *et al.*, 1993). A corresponding reaction between the *p*-tolyl phosphine P(*p*-tol)_3_ and RhCl(cod)(THP) has now similarly led to formation of trace amounts of *trans*-RhCl(CO)[P(*p*-tol)_3_]_2_ that was identified by an X-ray structure as an acetone solvated species. The complex has been synthesized previously in high yield from RhCl_3_.3H_2_O (Evans *et al.*, 1990), while the method first reported 50 years ago used [RhCl(CO)_2_]_2_ as the precursor (Vallarino, 1957). Our structure is a further example of the 125 or so of the type with a *trans*-RhCl(CO) moiety associated with two *trans* phosphorus donor atoms (Cambridge Crystallography Data Base).

### Experimental

General. The RhCl(cod)(THP) precursor complex was synthesized by our reported method (Lorenzini *et al.*, 2007*a*); P(*p*-tol)_3_ (a Strem Chemicals product), and the deuterated solvents (Cambridge Isotope Laboratory) were used as received. The reaction between these reagents was performed under Ar or H_2_ using standard Schlenk techniques. ^31^P{^1^H}-NMR spectra were measured in acetone-*d*_6_/CD_3_OD at room temperature (~300 K) on a Bruker AV400 spectrometer, relative to external 85% aq H_3_PO_4_.

*trans*-RhCl(CO)[P(*p*-tol)_3_]_2_.(CH_3_)_2_CO. P(*p*-tol)_3_ (18.3 µl, 0.059 mmol) in acetone-*d*_6_ (0.3 ml) was added to a yellow CD_3_OD solution (0.3 ml) of RhCl(cod)(THP) (10.2 mg, 0.028 mmol) at room temperature under Ar to give rapid formation of a brown solution. Replacement of the Ar by H_2_ and subsequent shaking of the vessel resulted in a yellow solution. Over 12 h, a minute quantity of X-ray quality, yellow prism crystals of *trans*-RhCl(CO)[P(*p*-tol)_3_]_2_.(CH_3_)_2_CO deposited from the solution; the ^31^P{^1^H} spectrum of the solution revealed a complex mixture of species.

### Refinement

H atoms were placed in calculated positions [C—H = 0.95 Å (aromatic) and 0.98 Å (methyl)] and refined using a riding-model approximation, with *U*_iso_(H) = 1.2_eq_(C) and 1.5_eq_(C_methyl_).

### Crystal data

**Table:**

[RhCl(C_21_H_21_P)_2_(CO)]·C_3_H_6_O	*Z* = 2
*M**_r_* = 833.14	*F*_000_ = 864
Triclinic, *P*1	*D*_x_ = 1.308 Mg m^−^^3^
Hall symbol: -P 1	Mo *K*α radiation λ = 0.71073 Å
*a* = 10.784 (2) Å	Cell parameters from 5165 reflections
*b* = 12.859 (3) Å	θ = 2.5–25.0º
*c* = 17.086 (3) Å	µ = 0.58 mm^−^^1^
α = 70.852 (7)º	*T* = 173 (2) K
β = 84.790 (7)º	Prism, yellow
γ = 71.012 (6)º	0.25 × 0.10 × 0.07 mm
*V* = 2116.2 (7) Å^3^	

### Data collection

**Table:**

Bruker X8 APEXII diffractometer	9909 independent reflections
Radiation source: fine-focus sealed tube	6378 reflections with *I* > 2σ(*I*)
Monochromator: graphite	*R*_int_ = 0.063
*T* = 173(2) K	θ_max_ = 27.8º
area detector scans	θ_min_ = 1.8º
Absorption correction: multi-scan(SADABS; Bruker, 2003)	*h* = −14→14
*T*_min_ = 0.683, *T*_max_ = 0.960	*k* = −16→16
31662 measured reflections	*l* = −17→22

### Refinement

**Table:**

Refinement on *F*^2^	Secondary atom site location: difference Fourier map
Least-squares matrix: full	Hydrogen site location: inferred from neighbouring sites
*R*[*F*^2^ > 2σ(*F*^2^)] = 0.047	H-atom parameters constrained
*wR*(*F*^2^) = 0.115	*w* = 1/[σ^2^(*F*_o_^2^) + (0.045*P*)^2^] where *P* = (*F*_o_^2^ + 2*F*_c_^2^)/3
*S* = 1.00	(Δ/σ)_max_ = 0.008
9909 reflections	Δρ_max_ = 0.48 e Å^−^^3^
477 parameters	Δρ_min_ = −0.44 e Å^−^^3^
Primary atom site location: structure-invariant direct methods	Extinction correction: none

### Special details

**Table:**

Experimental. The molecule crystallizes with one molecule of acetone in the asymmetric unit.
Geometry. All e.s.d.'s (except the e.s.d. in the dihedral angle between two l.s. planes) are estimated using the full covariance matrix. The cell e.s.d.'s are taken into account individually in the estimation of e.s.d.'s in distances, angles and torsion angles; correlations between e.s.d.'s in cell parameters are only used when they are defined by crystal symmetry. An approximate (isotropic) treatment of cell e.s.d.'s is used for estimating e.s.d.'s involving l.s. planes.
Refinement. Refinement of *F*^2^ against ALL reflections. The weighted *R*-factor *wR* and goodness of fit *S* are based on *F*^2^, conventional *R*-factors *R* are based on *F*, with *F* set to zero for negative *F*^2^. The threshold expression of *F*^2^ > 2σ(*F*^2^) is used only for calculating *R*-factors(gt) *etc.* and is not relevant to the choice of reflections for refinement. *R*-factors based on *F*^2^ are statistically about twice as large as those based on *F*, and *R*-factors based on ALL data will be even larger.

### Fractional atomic coordinates and isotropic or equivalent isotropic displacement parameters (Å^2^)

**Table:**

	*x*	*y*	*z*	*U*_iso_*/*U*_eq_	
C1	0.5990 (3)	0.2216 (3)	0.5635 (2)	0.0401 (8)	
C2	0.5919 (4)	0.2248 (4)	0.4811 (2)	0.0593 (11)	
H2	0.6541	0.2482	0.4422	0.071*	
C3	0.4926 (4)	0.1932 (4)	0.4566 (3)	0.0626 (12)	
H3	0.4885	0.1961	0.4006	0.075*	
C4	0.4011 (4)	0.1583 (3)	0.5109 (3)	0.0500 (10)	
C5	0.4086 (4)	0.1555 (3)	0.5919 (2)	0.0497 (9)	
H5	0.3462	0.1316	0.6305	0.060*	
C6	0.5058 (4)	0.1871 (3)	0.6184 (2)	0.0444 (9)	
H6	0.5082	0.1850	0.6743	0.053*	
C7	0.2945 (4)	0.1269 (4)	0.4820 (3)	0.0721 (13)	
H7A	0.2363	0.1959	0.4424	0.108*	
H7B	0.2439	0.0969	0.5297	0.108*	
H7C	0.3339	0.0673	0.4552	0.108*	
C8	0.8679 (3)	0.1195 (3)	0.6269 (2)	0.0376 (8)	
C9	0.9652 (4)	0.1031 (3)	0.6818 (2)	0.0524 (10)	
H9	0.9593	0.1624	0.7048	0.063*	
C10	1.0716 (4)	0.0011 (4)	0.7039 (2)	0.0647 (12)	
H10	1.1376	−0.0074	0.7408	0.078*	
C11	1.0815 (4)	−0.0885 (3)	0.6721 (2)	0.0580 (11)	
C12	0.9877 (4)	−0.0710 (3)	0.6161 (2)	0.0568 (11)	
H12	0.9946	−0.1297	0.5923	0.068*	
C13	0.8816 (4)	0.0319 (3)	0.5932 (2)	0.0482 (9)	
H13	0.8182	0.0417	0.5540	0.058*	
C14	1.1942 (5)	−0.2021 (4)	0.6995 (3)	0.0964 (19)	
H14A	1.1901	−0.2389	0.7593	0.145*	
H14B	1.2779	−0.1862	0.6863	0.145*	
H14C	1.1870	−0.2541	0.6703	0.145*	
C15	0.7857 (3)	0.3500 (3)	0.5080 (2)	0.0384 (8)	
C16	0.6944 (4)	0.4489 (3)	0.4564 (2)	0.0509 (9)	
H16	0.6032	0.4585	0.4624	0.061*	
C17	0.7368 (4)	0.5328 (3)	0.3964 (2)	0.0555 (11)	
H17	0.6734	0.5988	0.3619	0.067*	
C18	0.8699 (4)	0.5233 (3)	0.3852 (2)	0.0502 (10)	
C19	0.9593 (4)	0.4223 (3)	0.4345 (2)	0.0537 (10)	
H19	1.0505	0.4112	0.4270	0.064*	
C20	0.9183 (4)	0.3373 (3)	0.4947 (2)	0.0453 (9)	
H20	0.9820	0.2694	0.5271	0.054*	
C21	0.9137 (5)	0.6199 (3)	0.3232 (3)	0.0717 (13)	
H21A	1.0019	0.6136	0.3385	0.107*	
H21B	0.8525	0.6952	0.3234	0.107*	
H21C	0.9151	0.6130	0.2676	0.107*	
C22	0.4778 (3)	0.4089 (2)	0.8594 (2)	0.0339 (7)	
C23	0.3779 (4)	0.4611 (3)	0.7984 (2)	0.0450 (9)	
H23	0.3992	0.4906	0.7419	0.054*	
C24	0.2485 (4)	0.4700 (3)	0.8197 (2)	0.0499 (9)	
H24	0.1825	0.5066	0.7774	0.060*	
C25	0.2130 (4)	0.4271 (3)	0.9009 (3)	0.0510 (10)	
C26	0.3112 (4)	0.3759 (3)	0.9616 (2)	0.0513 (10)	
H26	0.2888	0.3466	1.0178	0.062*	
C27	0.4431 (4)	0.3664 (3)	0.9419 (2)	0.0434 (9)	
H27	0.5085	0.3311	0.9845	0.052*	
C28	0.0725 (4)	0.4330 (4)	0.9218 (3)	0.0729 (13)	
H28A	0.0158	0.5136	0.9009	0.109*	
H28B	0.0643	0.4036	0.9821	0.109*	
H28C	0.0459	0.3855	0.8961	0.109*	
C29	0.6443 (3)	0.5526 (2)	0.80580 (19)	0.0338 (7)	
C30	0.5354 (4)	0.6404 (3)	0.8164 (2)	0.0430 (9)	
H30	0.4555	0.6245	0.8342	0.052*	
C31	0.5418 (4)	0.7522 (3)	0.8012 (2)	0.0520 (10)	
H31	0.4655	0.8117	0.8075	0.062*	
C32	0.6576 (4)	0.7778 (3)	0.7771 (2)	0.0489 (10)	
C33	0.7671 (4)	0.6893 (3)	0.7667 (2)	0.0543 (10)	
H33	0.8474	0.7050	0.7498	0.065*	
C34	0.7607 (4)	0.5780 (3)	0.7807 (2)	0.0462 (9)	
H34	0.8364	0.5189	0.7730	0.055*	
C35	0.6639 (5)	0.8986 (3)	0.7602 (3)	0.0728 (14)	
H35A	0.6054	0.9521	0.7133	0.109*	
H35B	0.7540	0.8992	0.7469	0.109*	
H35C	0.6363	0.9230	0.8094	0.109*	
C36	0.7593 (3)	0.3203 (3)	0.91027 (19)	0.0358 (8)	
C37	0.7520 (3)	0.3574 (3)	0.9795 (2)	0.0404 (8)	
H37	0.6827	0.4237	0.9833	0.048*	
C38	0.8462 (4)	0.2972 (3)	1.0428 (2)	0.0495 (9)	
H38	0.8390	0.3224	1.0899	0.059*	
C39	0.9505 (4)	0.2012 (3)	1.0386 (2)	0.0546 (10)	
C40	0.9580 (4)	0.1654 (3)	0.9697 (2)	0.0545 (10)	
H40	1.0287	0.1001	0.9658	0.065*	
C41	0.8637 (3)	0.2234 (3)	0.9056 (2)	0.0445 (9)	
H41	0.8706	0.1971	0.8590	0.053*	
C42	1.0588 (5)	0.1410 (4)	1.1040 (3)	0.0855 (16)	
H42A	1.0831	0.0569	1.1159	0.128*	
H42B	1.0281	0.1605	1.1547	0.128*	
H42C	1.1354	0.1665	1.0837	0.128*	
C43	0.7044 (4)	0.4689 (3)	0.6425 (2)	0.0429 (8)	
C44	0.3191 (5)	0.0384 (4)	0.8769 (3)	0.0736 (13)	
C45	0.3625 (8)	−0.0618 (5)	0.9469 (4)	0.163 (3)	
H45A	0.3399	−0.1256	0.9391	0.245*	
H45B	0.3198	−0.0453	0.9966	0.245*	
H45C	0.4578	−0.0836	0.9535	0.245*	
C46	0.3191 (6)	0.1494 (4)	0.8789 (4)	0.122 (2)	
H46A	0.3996	0.1644	0.8540	0.183*	
H46B	0.3148	0.1493	0.9365	0.183*	
H46C	0.2428	0.2101	0.8476	0.183*	
O1	0.7199 (3)	0.5543 (2)	0.60038 (16)	0.0644 (8)	
O2	0.2868 (6)	0.0305 (5)	0.8141 (3)	0.174 (2)	
P1	0.73025 (9)	0.25561 (7)	0.60066 (5)	0.0366 (2)	
P2	0.64329 (8)	0.40376 (6)	0.82266 (5)	0.0322 (2)	
Cl1	0.65594 (10)	0.15903 (7)	0.80268 (5)	0.0504 (2)	
Rh1	0.68398 (3)	0.33396 (2)	0.710084 (16)	0.03563 (9)	

### Atomic displacement parameters (Å^2^)

**Table:**

	*U*^11^	*U*^22^	*U*^33^	*U*^12^	*U*^13^	*U*^23^
C1	0.042 (2)	0.0352 (18)	0.042 (2)	−0.0043 (16)	−0.0072 (17)	−0.0173 (16)
C2	0.058 (3)	0.082 (3)	0.049 (2)	−0.023 (2)	0.005 (2)	−0.036 (2)
C3	0.062 (3)	0.083 (3)	0.056 (3)	−0.021 (2)	−0.010 (2)	−0.039 (2)
C4	0.045 (2)	0.042 (2)	0.064 (3)	−0.0063 (18)	−0.015 (2)	−0.0221 (19)
C5	0.048 (2)	0.0373 (19)	0.060 (3)	−0.0108 (17)	−0.0059 (19)	−0.0121 (18)
C6	0.052 (2)	0.0347 (18)	0.045 (2)	−0.0094 (17)	−0.0073 (18)	−0.0127 (16)
C7	0.061 (3)	0.071 (3)	0.093 (3)	−0.018 (2)	−0.026 (3)	−0.032 (3)
C8	0.041 (2)	0.0357 (18)	0.0349 (19)	−0.0133 (15)	0.0020 (16)	−0.0092 (15)
C9	0.059 (3)	0.050 (2)	0.050 (2)	−0.014 (2)	−0.009 (2)	−0.0178 (19)
C10	0.052 (3)	0.074 (3)	0.050 (3)	−0.004 (2)	−0.014 (2)	−0.008 (2)
C11	0.058 (3)	0.051 (2)	0.042 (2)	0.002 (2)	0.007 (2)	−0.0048 (19)
C12	0.073 (3)	0.037 (2)	0.051 (2)	−0.004 (2)	0.009 (2)	−0.0171 (18)
C13	0.057 (2)	0.044 (2)	0.044 (2)	−0.0122 (19)	−0.0021 (18)	−0.0185 (17)
C14	0.092 (4)	0.081 (3)	0.060 (3)	0.030 (3)	0.008 (3)	−0.008 (3)
C15	0.045 (2)	0.0369 (18)	0.0330 (19)	−0.0080 (16)	−0.0008 (16)	−0.0153 (15)
C16	0.047 (2)	0.056 (2)	0.039 (2)	−0.0055 (19)	0.0022 (18)	−0.0115 (18)
C17	0.072 (3)	0.042 (2)	0.035 (2)	−0.001 (2)	−0.004 (2)	−0.0052 (17)
C18	0.069 (3)	0.039 (2)	0.042 (2)	−0.018 (2)	0.002 (2)	−0.0110 (17)
C19	0.052 (2)	0.047 (2)	0.058 (3)	−0.0141 (19)	0.003 (2)	−0.014 (2)
C20	0.051 (2)	0.0350 (18)	0.044 (2)	−0.0099 (17)	−0.0031 (18)	−0.0073 (16)
C21	0.096 (4)	0.054 (3)	0.061 (3)	−0.028 (3)	0.004 (3)	−0.008 (2)
C22	0.044 (2)	0.0234 (15)	0.0396 (19)	−0.0116 (14)	−0.0024 (16)	−0.0148 (14)
C23	0.053 (2)	0.046 (2)	0.042 (2)	−0.0219 (18)	−0.0039 (18)	−0.0140 (17)
C24	0.046 (2)	0.048 (2)	0.059 (3)	−0.0163 (18)	−0.0058 (19)	−0.0172 (19)
C25	0.051 (2)	0.048 (2)	0.067 (3)	−0.0218 (19)	0.011 (2)	−0.032 (2)
C26	0.061 (3)	0.048 (2)	0.050 (2)	−0.024 (2)	0.009 (2)	−0.0179 (19)
C27	0.058 (2)	0.0387 (19)	0.039 (2)	−0.0208 (18)	−0.0018 (18)	−0.0134 (16)
C28	0.061 (3)	0.086 (3)	0.090 (3)	−0.037 (3)	0.020 (3)	−0.042 (3)
C29	0.046 (2)	0.0251 (15)	0.0323 (18)	−0.0110 (15)	−0.0060 (15)	−0.0097 (13)
C30	0.049 (2)	0.0307 (17)	0.052 (2)	−0.0097 (16)	−0.0042 (18)	−0.0186 (16)
C31	0.063 (3)	0.0278 (17)	0.064 (3)	−0.0041 (18)	−0.014 (2)	−0.0193 (17)
C32	0.073 (3)	0.0269 (17)	0.048 (2)	−0.0181 (19)	−0.022 (2)	−0.0059 (16)
C33	0.064 (3)	0.041 (2)	0.064 (3)	−0.029 (2)	−0.009 (2)	−0.0082 (19)
C34	0.046 (2)	0.0339 (18)	0.061 (2)	−0.0130 (17)	−0.0028 (18)	−0.0166 (17)
C35	0.103 (4)	0.0286 (19)	0.087 (3)	−0.024 (2)	−0.035 (3)	−0.006 (2)
C36	0.044 (2)	0.0261 (16)	0.0368 (19)	−0.0108 (15)	−0.0026 (15)	−0.0084 (14)
C37	0.047 (2)	0.0356 (18)	0.038 (2)	−0.0117 (16)	−0.0026 (17)	−0.0117 (15)
C38	0.059 (3)	0.054 (2)	0.037 (2)	−0.021 (2)	−0.0058 (18)	−0.0114 (18)
C39	0.054 (3)	0.050 (2)	0.053 (2)	−0.017 (2)	−0.011 (2)	−0.0023 (19)
C40	0.052 (2)	0.0330 (19)	0.069 (3)	−0.0040 (18)	−0.006 (2)	−0.0101 (19)
C41	0.047 (2)	0.0332 (18)	0.054 (2)	−0.0105 (17)	−0.0046 (18)	−0.0162 (17)
C42	0.077 (3)	0.093 (4)	0.064 (3)	−0.014 (3)	−0.030 (3)	0.001 (3)
C43	0.053 (2)	0.0378 (19)	0.040 (2)	−0.0105 (17)	0.0023 (17)	−0.0192 (17)
C44	0.086 (4)	0.065 (3)	0.079 (3)	−0.020 (3)	−0.011 (3)	−0.035 (3)
C45	0.255 (10)	0.069 (4)	0.099 (5)	−0.003 (5)	0.012 (5)	0.008 (4)
C46	0.131 (5)	0.068 (4)	0.176 (6)	−0.039 (4)	0.006 (5)	−0.044 (4)
O1	0.098 (2)	0.0425 (15)	0.0528 (17)	−0.0303 (16)	0.0102 (16)	−0.0095 (13)
O2	0.221 (6)	0.186 (5)	0.152 (5)	−0.071 (4)	−0.059 (4)	−0.078 (4)
P1	0.0443 (5)	0.0336 (5)	0.0346 (5)	−0.0106 (4)	−0.0017 (4)	−0.0153 (4)
P2	0.0424 (5)	0.0236 (4)	0.0335 (5)	−0.0105 (4)	−0.0028 (4)	−0.0116 (3)
Cl1	0.0841 (7)	0.0353 (4)	0.0416 (5)	−0.0291 (5)	0.0002 (5)	−0.0145 (4)
Rh1	0.04978 (18)	0.02744 (14)	0.03398 (16)	−0.01380 (12)	0.00046 (12)	−0.01354 (11)

### Geometric parameters (Å, °)

**Table:**

C1—C6	1.390 (5)	C25—C26	1.389 (5)
C1—C2	1.403 (5)	C25—C28	1.508 (5)
C1—P1	1.830 (3)	C26—C27	1.407 (5)
C2—C3	1.401 (5)	C26—H26	0.95
C2—H2	0.95	C27—H27	0.95
C3—C4	1.373 (5)	C28—H28A	0.98
C3—H3	0.95	C28—H28B	0.98
C4—C5	1.382 (5)	C28—H28C	0.98
C4—C7	1.505 (5)	C29—C30	1.386 (4)
C5—C6	1.397 (5)	C29—C34	1.395 (5)
C5—H5	0.95	C29—P2	1.843 (3)
C6—H6	0.95	C30—C31	1.399 (4)
C7—H7A	0.98	C30—H30	0.95
C7—H7B	0.98	C31—C32	1.387 (5)
C7—H7C	0.98	C31—H31	0.95
C8—C13	1.387 (4)	C32—C33	1.394 (5)
C8—C9	1.389 (5)	C32—C35	1.506 (4)
C8—P1	1.840 (3)	C33—C34	1.396 (4)
C9—C10	1.397 (5)	C33—H33	0.95
C9—H9	0.95	C34—H34	0.95
C10—C11	1.399 (6)	C35—H35A	0.98
C10—H10	0.95	C35—H35B	0.98
C11—C12	1.368 (5)	C35—H35C	0.98
C11—C14	1.526 (5)	C36—C37	1.400 (4)
C12—C13	1.404 (5)	C36—C41	1.401 (4)
C12—H12	0.95	C36—P2	1.836 (3)
C13—H13	0.95	C37—C38	1.392 (5)
C14—H14A	0.98	C37—H37	0.95
C14—H14B	0.98	C38—C39	1.391 (5)
C14—H14C	0.98	C38—H38	0.95
C15—C20	1.391 (5)	C39—C40	1.385 (5)
C15—C16	1.405 (5)	C39—C42	1.515 (5)
C15—P1	1.837 (3)	C40—C41	1.401 (5)
C16—C17	1.391 (5)	C40—H40	0.95
C16—H16	0.95	C41—H41	0.95
C17—C18	1.400 (5)	C42—H42A	0.98
C17—H17	0.95	C42—H42B	0.98
C18—C19	1.393 (5)	C42—H42C	0.98
C18—C21	1.523 (5)	C43—O1	1.154 (4)
C19—C20	1.394 (5)	C43—Rh1	1.812 (4)
C19—H19	0.95	C44—O2	1.203 (6)
C20—H20	0.95	C44—C45	1.417 (6)
C21—H21A	0.98	C44—C46	1.438 (6)
C21—H21B	0.98	C45—H45A	0.98
C21—H21C	0.98	C45—H45B	0.98
C22—C27	1.398 (4)	C45—H45C	0.98
C22—C23	1.405 (5)	C46—H46A	0.98
C22—P2	1.825 (3)	C46—H46B	0.98
C23—C24	1.387 (5)	C46—H46C	0.98
C23—H23	0.95	P1—Rh1	2.3449 (9)
C24—C25	1.385 (5)	P2—Rh1	2.3283 (9)
C24—H24	0.95	Cl1—Rh1	2.3822 (9)
			
C6—C1—C2	118.3 (3)	C26—C27—H27	120.0
C6—C1—P1	119.8 (3)	C25—C28—H28A	109.5
C2—C1—P1	121.9 (3)	C25—C28—H28B	109.5
C3—C2—C1	119.7 (4)	H28A—C28—H28B	109.5
C3—C2—H2	120.2	C25—C28—H28C	109.5
C1—C2—H2	120.2	H28A—C28—H28C	109.5
C4—C3—C2	122.1 (4)	H28B—C28—H28C	109.5
C4—C3—H3	119.0	C30—C29—C34	118.4 (3)
C2—C3—H3	119.0	C30—C29—P2	123.4 (3)
C3—C4—C5	117.9 (3)	C34—C29—P2	118.1 (2)
C3—C4—C7	120.6 (4)	C29—C30—C31	120.7 (3)
C5—C4—C7	121.5 (4)	C29—C30—H30	119.7
C4—C5—C6	121.5 (4)	C31—C30—H30	119.7
C4—C5—H5	119.2	C32—C31—C30	121.2 (3)
C6—C5—H5	119.2	C32—C31—H31	119.4
C1—C6—C5	120.5 (3)	C30—C31—H31	119.4
C1—C6—H6	119.7	C31—C32—C33	118.0 (3)
C5—C6—H6	119.7	C31—C32—C35	121.1 (4)
C4—C7—H7A	109.5	C33—C32—C35	120.9 (4)
C4—C7—H7B	109.5	C32—C33—C34	121.0 (4)
H7A—C7—H7B	109.5	C32—C33—H33	119.5
C4—C7—H7C	109.5	C34—C33—H33	119.5
H7A—C7—H7C	109.5	C29—C34—C33	120.6 (3)
H7B—C7—H7C	109.5	C29—C34—H34	119.7
C13—C8—C9	117.6 (3)	C33—C34—H34	119.7
C13—C8—P1	123.2 (3)	C32—C35—H35A	109.5
C9—C8—P1	119.2 (3)	C32—C35—H35B	109.5
C8—C9—C10	121.4 (4)	H35A—C35—H35B	109.5
C8—C9—H9	119.3	C32—C35—H35C	109.5
C10—C9—H9	119.3	H35A—C35—H35C	109.5
C9—C10—C11	120.4 (4)	H35B—C35—H35C	109.5
C9—C10—H10	119.8	C37—C36—C41	118.8 (3)
C11—C10—H10	119.8	C37—C36—P2	121.0 (2)
C12—C11—C10	118.1 (4)	C41—C36—P2	120.0 (3)
C12—C11—C14	121.6 (4)	C38—C37—C36	120.1 (3)
C10—C11—C14	120.4 (4)	C38—C37—H37	119.9
C11—C12—C13	121.5 (4)	C36—C37—H37	119.9
C11—C12—H12	119.2	C39—C38—C37	121.5 (3)
C13—C12—H12	119.2	C39—C38—H38	119.2
C8—C13—C12	120.9 (3)	C37—C38—H38	119.2
C8—C13—H13	119.6	C40—C39—C38	118.2 (3)
C12—C13—H13	119.6	C40—C39—C42	119.9 (4)
C11—C14—H14A	109.5	C38—C39—C42	121.8 (4)
C11—C14—H14B	109.5	C39—C40—C41	121.5 (3)
H14A—C14—H14B	109.5	C39—C40—H40	119.2
C11—C14—H14C	109.5	C41—C40—H40	119.2
H14A—C14—H14C	109.5	C36—C41—C40	119.8 (3)
H14B—C14—H14C	109.5	C36—C41—H41	120.1
C20—C15—C16	117.9 (3)	C40—C41—H41	120.1
C20—C15—P1	121.3 (3)	C39—C42—H42A	109.5
C16—C15—P1	120.0 (3)	C39—C42—H42B	109.5
C17—C16—C15	120.2 (4)	H42A—C42—H42B	109.5
C17—C16—H16	119.9	C39—C42—H42C	109.5
C15—C16—H16	119.9	H42A—C42—H42C	109.5
C16—C17—C18	122.2 (3)	H42B—C42—H42C	109.5
C16—C17—H17	118.9	O1—C43—Rh1	178.5 (3)
C18—C17—H17	118.9	O2—C44—C45	120.1 (5)
C19—C18—C17	116.7 (3)	O2—C44—C46	119.4 (6)
C19—C18—C21	121.9 (4)	C45—C44—C46	120.5 (5)
C17—C18—C21	121.3 (4)	C44—C45—H45A	109.5
C18—C19—C20	121.7 (4)	C44—C45—H45B	109.5
C18—C19—H19	119.2	H45A—C45—H45B	109.5
C20—C19—H19	119.2	C44—C45—H45C	109.5
C15—C20—C19	121.2 (3)	H45A—C45—H45C	109.5
C15—C20—H20	119.4	H45B—C45—H45C	109.5
C19—C20—H20	119.4	C44—C46—H46A	109.5
C18—C21—H21A	109.5	C44—C46—H46B	109.5
C18—C21—H21B	109.5	H46A—C46—H46B	109.5
H21A—C21—H21B	109.5	C44—C46—H46C	109.5
C18—C21—H21C	109.5	H46A—C46—H46C	109.5
H21A—C21—H21C	109.5	H46B—C46—H46C	109.5
H21B—C21—H21C	109.5	C1—P1—C15	105.08 (16)
C27—C22—C23	118.2 (3)	C1—P1—C8	104.96 (15)
C27—C22—P2	125.8 (3)	C15—P1—C8	103.74 (15)
C23—C22—P2	115.9 (3)	C1—P1—Rh1	117.85 (12)
C24—C23—C22	120.6 (3)	C15—P1—Rh1	112.37 (10)
C24—C23—H23	119.7	C8—P1—Rh1	111.61 (11)
C22—C23—H23	119.7	C22—P2—C36	108.70 (15)
C25—C24—C23	121.8 (4)	C22—P2—C29	102.88 (14)
C25—C24—H24	119.1	C36—P2—C29	101.69 (14)
C23—C24—H24	119.1	C22—P2—Rh1	109.37 (10)
C24—C25—C26	117.9 (4)	C36—P2—Rh1	115.03 (11)
C24—C25—C28	120.6 (4)	C29—P2—Rh1	118.15 (11)
C26—C25—C28	121.5 (4)	C43—Rh1—P2	91.43 (10)
C25—C26—C27	121.6 (4)	C43—Rh1—P1	90.55 (10)
C25—C26—H26	119.2	P2—Rh1—P1	177.46 (3)
C27—C26—H26	119.2	C43—Rh1—Cl1	178.12 (10)
C22—C27—C26	119.9 (3)	P2—Rh1—Cl1	86.69 (3)
C22—C27—H27	120.0	P1—Rh1—Cl1	91.33 (3)
			
C6—C1—C2—C3	−0.2 (6)	C37—C38—C39—C42	175.2 (4)
P1—C1—C2—C3	177.3 (3)	C38—C39—C40—C41	−0.1 (6)
C1—C2—C3—C4	−0.3 (6)	C42—C39—C40—C41	−176.1 (4)
C2—C3—C4—C5	0.4 (6)	C37—C36—C41—C40	0.2 (5)
C2—C3—C4—C7	179.0 (4)	P2—C36—C41—C40	174.9 (3)
C3—C4—C5—C6	0.1 (5)	C39—C40—C41—C36	0.4 (6)
C7—C4—C5—C6	−178.6 (3)	C6—C1—P1—C15	−157.5 (3)
C2—C1—C6—C5	0.6 (5)	C2—C1—P1—C15	25.0 (3)
P1—C1—C6—C5	−176.9 (2)	C6—C1—P1—C8	93.4 (3)
C4—C5—C6—C1	−0.5 (5)	C2—C1—P1—C8	−84.0 (3)
C13—C8—C9—C10	−1.4 (6)	C6—C1—P1—Rh1	−31.5 (3)
P1—C8—C9—C10	179.8 (3)	C2—C1—P1—Rh1	151.1 (3)
C8—C9—C10—C11	−1.2 (6)	C20—C15—P1—C1	−138.6 (3)
C9—C10—C11—C12	3.0 (6)	C16—C15—P1—C1	52.1 (3)
C9—C10—C11—C14	−177.1 (4)	C20—C15—P1—C8	−28.7 (3)
C10—C11—C12—C13	−2.3 (6)	C16—C15—P1—C8	162.0 (3)
C14—C11—C12—C13	177.7 (4)	C20—C15—P1—Rh1	92.0 (3)
C9—C8—C13—C12	2.1 (5)	C16—C15—P1—Rh1	−77.3 (3)
P1—C8—C13—C12	−179.2 (3)	C13—C8—P1—C1	23.0 (3)
C11—C12—C13—C8	−0.2 (6)	C9—C8—P1—C1	−158.4 (3)
C20—C15—C16—C17	−2.6 (5)	C13—C8—P1—C15	−87.1 (3)
P1—C15—C16—C17	167.0 (3)	C9—C8—P1—C15	91.6 (3)
C15—C16—C17—C18	−0.4 (6)	C13—C8—P1—Rh1	151.7 (3)
C16—C17—C18—C19	3.0 (5)	C9—C8—P1—Rh1	−29.6 (3)
C16—C17—C18—C21	−175.8 (3)	C27—C22—P2—C36	5.7 (3)
C17—C18—C19—C20	−2.7 (5)	C23—C22—P2—C36	−174.6 (2)
C21—C18—C19—C20	176.1 (3)	C27—C22—P2—C29	−101.5 (3)
C16—C15—C20—C19	2.9 (5)	C23—C22—P2—C29	78.1 (3)
P1—C15—C20—C19	−166.6 (3)	C27—C22—P2—Rh1	132.1 (2)
C18—C19—C20—C15	−0.2 (5)	C23—C22—P2—Rh1	−48.3 (2)
C27—C22—C23—C24	0.0 (5)	C37—C36—P2—C22	−61.6 (3)
P2—C22—C23—C24	−179.7 (2)	C41—C36—P2—C22	123.7 (3)
C22—C23—C24—C25	−0.8 (5)	C37—C36—P2—C29	46.4 (3)
C23—C24—C25—C26	1.1 (5)	C41—C36—P2—C29	−128.2 (3)
C23—C24—C25—C28	−177.2 (3)	C37—C36—P2—Rh1	175.4 (2)
C24—C25—C26—C27	−0.7 (5)	C41—C36—P2—Rh1	0.8 (3)
C28—C25—C26—C27	177.6 (3)	C30—C29—P2—C22	−3.3 (3)
C23—C22—C27—C26	0.5 (5)	C34—C29—P2—C22	177.0 (3)
P2—C22—C27—C26	−179.9 (2)	C30—C29—P2—C36	−115.8 (3)
C25—C26—C27—C22	−0.1 (5)	C34—C29—P2—C36	64.5 (3)
C34—C29—C30—C31	0.8 (5)	C30—C29—P2—Rh1	117.3 (3)
P2—C29—C30—C31	−178.9 (3)	C34—C29—P2—Rh1	−62.4 (3)
C29—C30—C31—C32	−1.6 (6)	C22—P2—Rh1—C43	117.67 (16)
C30—C31—C32—C33	1.4 (6)	C36—P2—Rh1—C43	−119.70 (16)
C30—C31—C32—C35	179.5 (3)	C29—P2—Rh1—C43	0.54 (17)
C31—C32—C33—C34	−0.5 (6)	C22—P2—Rh1—Cl1	−62.38 (11)
C35—C32—C33—C34	−178.6 (4)	C36—P2—Rh1—Cl1	60.25 (12)
C30—C29—C34—C33	0.1 (5)	C29—P2—Rh1—Cl1	−179.51 (12)
P2—C29—C34—C33	179.8 (3)	C1—P1—Rh1—C43	−114.47 (17)
C32—C33—C34—C29	−0.2 (6)	C15—P1—Rh1—C43	7.91 (16)
C41—C36—C37—C38	−1.0 (5)	C8—P1—Rh1—C43	123.98 (16)
P2—C36—C37—C38	−175.7 (3)	C1—P1—Rh1—Cl1	65.64 (13)
C36—C37—C38—C39	1.3 (5)	C15—P1—Rh1—Cl1	−171.98 (12)
C37—C38—C39—C40	−0.7 (6)	C8—P1—Rh1—Cl1	−55.92 (12)

### Hydrogen-bond geometry (Å, °)

**Table:**

*D*—H···*A*	*D*—H	H···*A*	*D*···*A*	*D*—H···*A*
C10—H10···O2^i^	0.95	2.38	3.302 (8)	163
C46—H46A···Cl1	0.98	2.81	3.773 (7)	168

Symmetry codes: (i) *x*+1, *y*, *z*.

## References

[bb1] Altomare, A., Burla, M. C., Camalli, M., Cascarano, G. L., Giacovazzo, C., Guagliardi, A., Moliterni, A. G. G., Polidori, G. & Spagna, R. (1999). *J. Appl. Cryst.* **32**, 115–119.

[bb2] Beck, C. M., Rathmill, S. E., Park, Y. J., Chen, J., Crabtree, R. H., Liable-Sands, L. M. & Rheingold, A. L. (1999). *Organometallics*, **18**, 5311–5317.

[bb3] Bruker (2003). *SADABS* Version 2.10. Bruker AXS Inc., Madison, Wisconsin, USA.

[bb4] Bruker (2005). *SAINT* Version 7.23. Bruker AXS Inc., Madison, Wisconsin, USA.

[bb5] Bruker (2006). *APEX2* Version 2.10. Bruker AXS Inc., Madison, Wisconsin, USA.

[bb6] Evans, D., Osborn, J. A. & Wilkinson, G. (1990). *Inorg. Synth.* **28**, 79–80.

[bb7] Farrugia, L. J. (1997). *J. Appl. Cryst.* **30**, 565.

[bb8] Farrugia, L. J. (1999). *J. Appl. Cryst.* **32**, 837–838.

[bb9] Higham, L. J., Whittlesey, M. K. & Wood, P. T. (2004). *J. Chem. Soc. Dalton Trans.* pp. 4202–4208. 10.1039/b411701h 15573173

[bb10] Hoye, P. A. T., Pringle, P. G., Smith, M. B. & Worboys, K. (1993). *J. Chem. Soc. Dalton Trans.* pp. 269–274.

[bb11] Lorenzini, F., Patrick, B. O. & James, B. R. (2007*a*). *J. Chem. Soc. Dalton Trans.* pp. 3224–3226. 10.1039/b706691k 17893766

[bb12] Lorenzini, F., Patrick, B. O. & James, B. R. (2007*b*). *Inorg. Chem.* **46**, 8998–9002. 10.1021/ic7012182 17867681

[bb13] Lorenzini, F., Patrick, B. O. & James, B. R. (2008*a*). *Inorg. Chim. Acta*, doi: 10.1016/j.ica.2007.10.044.

[bb14] Lorenzini, F., Patrick, B. O. & James, B. R. (2008*b*). *Acta Cryst.* E**64**, m179–m180. 10.1107/S1600536807065877 PMC2915114 21200527

[bb15] Sheldrick, G. M. (2008). *Acta Cryst.* A**64**, 112–122. 10.1107/S0108767307043930 18156677

[bb16] Vallarino, L. (1957). *J. Chem. Soc.* pp. 2287–2292.

